# Accurate validation of ultrasound imaging of prostate cancer: a review of challenges in registration of imaging and histopathology

**DOI:** 10.1007/s40477-018-0311-8

**Published:** 2018-07-30

**Authors:** Rogier R. Wildeboer, Ruud J.G. van Sloun, Arnoud W. Postema, Christophe K. Mannaerts, Maudy Gayet, Harrie P. Beerlage, Hessel Wijkstra, Massimo Mischi

**Affiliations:** 1 Laboratory of Biomedical Diagnostics, Department of Electrical Engineering, Eindhoven University of Technology, De Rondom 70, 5612 AP Eindhoven, The Netherlands; 2 Department of Urology, Academic Medical Center University Hospital, Meibergdreef 9, 1105 AZ Amsterdam, The Netherlands; 3 Department of Urology, Jeroen Bosch Hospital, Henri Dunantstraat 1, 5223 GZ ‘S-Hertogenbosch, The Netherlands

**Keywords:** Prostate cancer, Diagnostic imaging, Validation studies as topic, Histology, Review

## Abstract

As the development of modalities for prostate cancer (PCa) imaging advances, the challenge of accurate registration between images and histopathologic ground truth becomes more pressing. Localization of PCa, rather than detection, requires a pixel-to-pixel validation of imaging based on histopathology after radical prostatectomy. Such a registration procedure is challenging for ultrasound modalities; not only the deformations of the prostate after resection have to be taken into account, but also the deformation due to the employed transrectal probe and the mismatch in orientation between imaging planes and pathology slices. In this work, we review the latest techniques to facilitate accurate validation of PCa localization in ultrasound imaging studies and extrapolate a general strategy for implementation of a registration procedure.

## SOMMARIO

Con lo sviluppo di nuove tecnologie di imaging per il cancro alla prostata, è diventata più pressante l'esigenza di una accurata registrazione e confronto tra le immagini e i reperti istopatologici. La localizzazione del tumore prostatico, oltre che il suo riconoscimento, richiede una validazione "pixel-topixel" dell'imaging basata sulla sua istopatologia ottenuta con la prostatectomia radicale. La tecnica di registrazione è cruciale per la metodica ecografica; devono essere tenute in conto non solo le deformazioni della prostata dopo resezione, ma anche la deformazione dovuta alla sonda transrettale impiegata e la possibile non corrispondenza tra i piani di orientamento impiegati nella formazione delle immagini e le sezioni istologiche. In questo lavoro gli Autori fanno una revisione delle recenti tecniche che facilitano la localizzazione del cancro della prostata in diagnostica ecografica ed estrapolano possibili strategie generali per implementare le procedure di registrazione degli esami.

## Introduction

Prostate cancer (PCa) imaging is a very active field in medical science. Even though PCa exhibits the highest cancer incidence among the American male population [1], reliable imaging methods are not yet available. As a consequence, systematic 10–12-core needle-biopsy still is the guideline-recommended diagnostic strategy [2], a procedure that is known to lead to under diagnosis, overtreatment and complications [3, 4]. Research groups around the world are, therefore, investing in the development of imaging tools that might facilitate targeted biopsy and ultimately replace the biopsy procedure altogether. In addition, focal therapies are emerging to avoid the severe side effects associated with radical treatment of PCa, increasing the need for reliable imaging for treatment planning, monitoring and follow-up [5].

The development of new imaging technologies requires rigorous validation with the histopathological ground truth. Although histopathology of the excised prostate specimen after radical prostatectomy (RP) is considered to be preferred to validate PCa localization [6, 7], most investigators have been using transperineal or transrectal biopsies as reference standard (see, e.g. meta-analyses for multiparametric Magnetic Resonance Imaging (mpMRI) [6, 7]; ultrasound (US) modalities [8, 9]; and Positron Emission Tomography (PET)/Computed Tomography (CT), [10]). When RP histopathology is available, validation is generally based on cognitive matching between image and histopathology. Although seemingly straightforward, this procedure can be difficult, is prone to errors, and requires many (invalid) underlying assumptions. Researchers are, therefore, forced to perform the validation in regions, quadrants, zones or the prostate as a whole [6–10]. For targeted biopsy and focal therapy, however, we should aim for tumour localization at a higher resolution.

Matching of images and histopathology is a challenge; the prostate deforms considerably after excision and pathological preparation and these substantial differences between in vivo and ex vivo shape must be compensated. In the past decades, many registration methods have been developed to map the ex vivo findings onto the in vivo images. For MRI, CT and PET, slice selection algorithms have been implemented to find the exact lesion locations in the image [11, 12]. In this respect, ultrasonic modalities are often overlooked, since their typical two-dimensional imaging planes are very differently oriented than the histopathology slices [13, 14]. Moreover, the manual pressure of the transrectal probe adds to the deformation between in vivo and ex vivo [13]. In this review, we survey the spectrum of available techniques and other important considerations for an accurate validation of ultrasonic techniques for prostate cancer imaging.

## General workflow

In general, pixel-to-pixel validation strategies require a standardized histopathology protocol (in which the histopathological data are assembled into a model), a registration procedure (in which deformations are compensated for) and a correlation step (in which the pathology-proven PCa lesions are superimposed onto the images). We review these steps sequentially.

### Step 1A: histopathological modelling

The standard pathology protocol comprises RP specimen fixation, sectioning in 2–4-mm thick slices, staining of front-faces and histopathologic examination of whole-mount or smaller sections [15, 16]. As previously mentioned, two-dimensional (2D) transrectal US imaging planes often have a very different orientation than the RP slices. An imaging plane can, therefore, only be accurately matched to histopathologic data by combining the information from all slices it crosses. Three-dimensional reconstruction and adequate interpolation of histopathology are, therefore, of vital importance [11, 17–19]. These models can also be readily used for the validation of three-dimensional (3D) US imaging solutions for B-mode, elastography and contrast-enhanced ultrasonography [20]. Paradoxically, validation of 3D imaging modalities is less dependent on a proper 3D histology model as their imaging is not bound two a particular 2D plane (i.e. one can select the voxels that correspond to the histological slice).

To construct a suitable histopathological model, one hugely relies on assumptions concerning slice location, orientation and deformations during the pathological work flow [21]. However, it has been reported that almost nine tenth of European pathologists section the prostate without using a special cutting device [22], which might lead to histopathologic slices not being parallel or of equal thickness [23]. In recent years, many groups developed slicing devices to standardize the sectioning process and minimize inaccuracies [24]. Still, it was quantified that microtome cutting exhibits standard deviations of 0.2–0.5 mm in ~ 4-mm thick slices and 0.9°–1.1° in inter-slice front-face orientation [21, 25, 26].

The conversion to three dimensions requires spatial alignment of the histopathologically annotated slices. Although manual alignment is most common [11], there are strategies involving the use of anatomical landmarks (e.g. [27]), block-face photographs taken during the sectioning process (e.g. [12, 21]) or mutual information-based intensity matching (e.g. [28]). As natural features and other (anatomical) information do not usually persist over multiple slicing distances, similarity-based alignment becomes increasingly difficult when using larger slice thicknesses [27]. Some authors introduce external fiducial markers to guide the alignment [27, 29, 30]. Naturally, lower reconstruction errors associated with more sophisticated techniques come at the price of the labour involved.

**Fig. 1:**
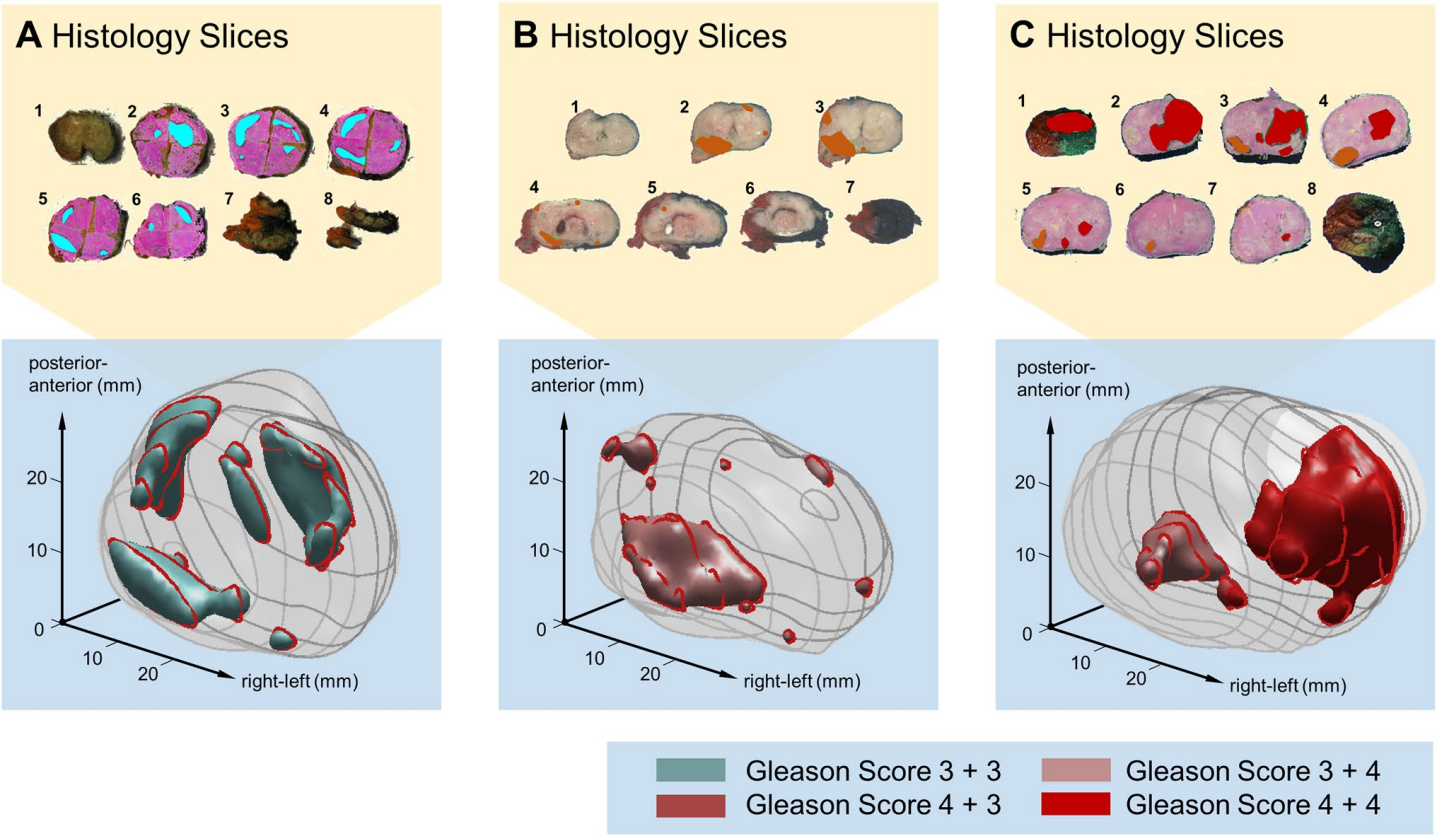
Three examples of 3D histopathology reconstructions from tumour-delineated macro-photos of the sliced radical prostatectomy specimen **a**–**c**. Volumetric lesions are colour-coded to depict their Gleason Score

There are many algorithms available to build volumetric structures from stacked 2D data. For PCa lesions, most reported are simply stacking the slices [12] and extrapolating the histopathologic data over the entire slice thickness [31, 32]. More sophisticated algorithms use radial-basis functions [33] or spline functions [29] to smoothly interpolate the histopathologic data between the slices. A comprehensive overview of techniques is listed in Table 1. Obviously, the accuracy of these methods relies to a great extent on the precision of slicing and alignment. In previously published work, we found that a standard clinical workflow would lead to a 1.5-mm error margin in tumour boundary location [33]. As an example, Fig. 1 features an illustration of the 3D models generated by this technique.

**Table 1:** 3D modelling of histopathology and imaging

Source(s)		Modality	Method	Validation method	Performance
[31]	Malone, 2014	Histology	3D stacking, interpolation over inter-slice thickness	–	
[33]	Wildeboer, 2017	Histology	Radial-basis functions	90th percentile surface deviation simulation	1.5 mm
[29]	Taylor, 2004	Histology	Spline interpolation of distance field [72] ex vivo US	Specimen volume accuracy	92% ± 3%.
[73]	Hughes, 2012	Histology	Stacking based on fiducial markers	Average deviation of ejactory ducts	1.5 mm,
[74]	Werahera, 1995	Histology	Linear inter-slice interpolation and extrapolation	Specimen volume accuracy	~ 4.5%
[75]	Xuan, 1997	Histology	Elastic contour interpolation	–	–
[75]	Xuan, 1997	Histology	Surface spine model	–	–
[76]	Tutar, 2004	Ultrasound	Fourier-description deformable models	–	–
[14]	Cool, 2006	Ultrasound	Radial-basis functions	Mean surface deviation simulation	1.34 ± 0.20 mm
[77]	Hibbard, 2012	Ultrasound	Shape-optimal RBFs implicit surface reconstruction	Mean surface deviation expert	< 0.5 mm

*RBF* radial-basis functions

Some studies make use of ex vivo imaging before slicing [12, 26, 34–37]. As an intermediate step, the histopathological data are mapped onto the deformed, ex vivo 3D model of the prostate prior to the registration to the in vivo shape. A comparison of registration with and without ex vivo MRI, however, did not show significant improvement [38]. This suggests that in vivo to ex vivo mapping is the crucial transformation. Moreover, even when ex vivo (US) scans are used for the histopathology reconstruction, this method would still require interpolation of the tumour delineations into tumour volumes.

### Step 1B: three-dimensional modelling of imaging

**Fig. 2:**
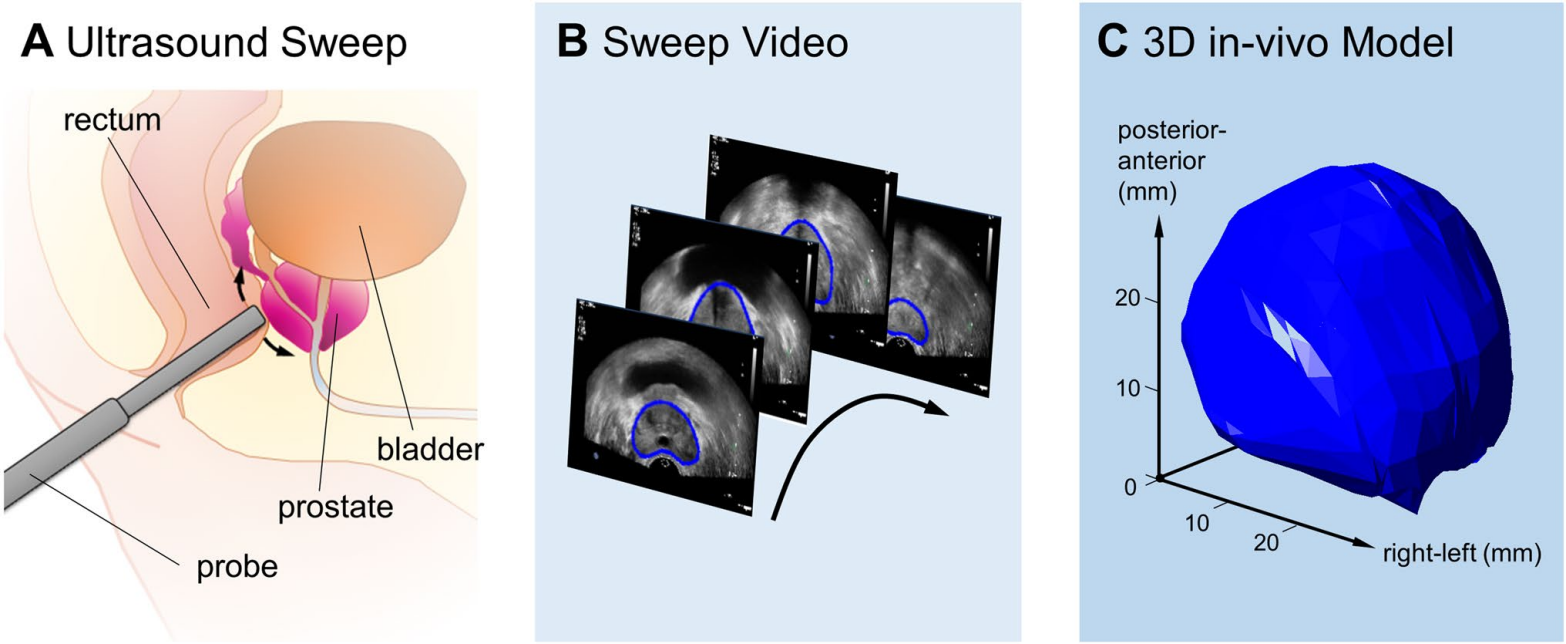
Example of in vivo 3D reconstruction of the prostate based on a 2D US sweep: **a** schematic of the sweep procedure, **b** representation of manually segmented prostate in the ultrasound sweep video, and **c** resulting 3D reconstruction

As we are interested in matching the in vivo and ex vivo prostate, a 3D model of the in vivo shape is also required. When 3D imaging is not available, such a model can be reconstructed from a 2D sweep (e.g. [13]) as shown in Fig. 2. The 2D images will have to be segmented and connected, for example using algorithms similar to those used for tumour interpolation (see Table 1). As described in Step 3, it is important to retrieve the location of the imaging plane of interest in this 3D model afterwards.

### Step 2: registration

Unfortunately, it is not possible to directly match the in vivo prostate to the reconstructed RP specimen, even when the ex vivo shape is perfectly restored. Mainly the loss of vascular pressure and the absence of the connective tissue encapsulation after removal from the body cause the prostate to deform after resection. Orzcyk et al. have shown that the prostate shrinks unevenly; on average, the prostate is 2.9% smaller in the base-apical direction, whereas it shrinks by 9.7% in the anterior–posterior direction [39]. The pathological preparation of the ex vivo specimen also has an effect (especially the formalin fixation [40] and cutting procedure [24]), whereas the in vivo shape might already have been changed by a filled bladder pressing onto the gland. Moreover, ultrasonic modalities alter the in vivo shape considerably by employing a transrectal probe. On top of that, there are indications of inhomogeneous deformation within the prostate due to differences in tissue elasticity between zones and between anatomical or pathological features [41, 42].

Registration algorithms are designed to digitally translate the deformed prostate back to its original form, ranging from rigid (i.e. only translation and rotation) to fully elastic methods. Many prostate-applicable algorithms have been developed for MRI, but these can often be readily applied to US. Techniques that only work in 2D, because they make use of prior MRI-slice to histopathology-slice matching [18, 26, 34, 43–47], will have to be expanded to 3D. Unfortunately, some of these methods, especially those using similarity measures, would then be seriously hampered by the low resolution in the longitudinal direction, requiring a slicing distance below the mm range.

It is important to note that registration techniques need guidance, that is, they require spatial correspondence information from both modalities to estimate the mapping from one to another. Conventionally, this is either intensity based or landmark based. For the latter approach, the prostatic capsule or (anatomical) landmarks are manually or automatically pinpointed in histopathology as well as imaging. The intensity-based approach does not need specific landmark pairs, but uses full-image similarity features such as correlation or mutual information between histopathology and imaging to guide the registration. As a next step, a warping algorithm interpolates the voxel-to-voxel displacement over the entire image. For this purpose, researchers have exploited everything from affine transformations (AT) (e.g. rotation, translation, scaling) up to elastic methods, based on basis-splines (BSp) [48] or thin-plate splines (TPS) [49].

A special case of registration is the use of image-based moulds [50–54]. This procedure requires a three-dimensional in vivo scan to fabricate a tailor-made mould to force the specimen into its in vivo shape during the sectioning process. Moulds can also be used to simulate a transrectal probe pressing against the specimen; however, for MRI, an endorectal-coil mimicking mould did not significantly improve the registration performance [55]. Obviously, moulding cannot take into account deformations within the prostate and cannot compensate for inhomogeneous shrinkage. The position and orientation of the prostate slices, on the other hand, are well controlled and easily recoverable.

In Table 2, a selection of registration methods applied in the prostate is listed. It is worth noting that the labour required substantially differs between registration procedures. Whereas semi-automated algorithms are easily manageable, protocols requiring manual delineation or ex vivo scans and fiducial marker placement are increasingly laborious. We also note that the performance of the various registration procedures is not verified in the same manner, making it difficult to compare the strategies; most articles quantify the error by the target registration error (TRE), but others mention the volumetric overlap or the result of visual inspection. Typically, only a relatively low number of prostatic specimens is used for the validation.

**Table 2:** List of registration algorithms used in the prostate

Source		Registration method			Verification				
Ref. Author, year		Guidance^a^	Warping^b^	Ex Vivo Scan	Method	Modality	2D/3D	#^c^	TRE^d^ (mm)
[78]	Zhan, 2007	Landmark-based: automatic	TPS	No	Manual landmarks	MRI	3D	5	0.82
[28]	Ou, 2009	Landmark-based: automatic	TPS	No	Manual landmarks	MRI	3D	5	0.79
[34]	Gibson, 2012	Landmark-based: ex vivo markers	AT	Yes	Manual ex vivo MRI landmarks	MRI	3D	9	0.71
[36]	Orczyk, 2012	Landmark-based: manual	AT	Yes	Manual landmarks	MRI	3D	3	1.59
[26]	Ward, 2012	Landmark-based: manual	TPS	Yes	Manual landmarks	MRI	2D	13	1.1
[38]	Orczyk, 2013	Landmark-based: manual	AT	No	Manual landmarks	MRI	3D	3	1.6
[46]	Commandeur, 2015	Landmark-based: manual (contours)	BSp	No	Manual landmarks	MRI	2D	3	4.9
[13]	Schalk, 2016	Landmark-based: manual (contours)	NN	No	Manual (PZ-TZ) landmarks	US	3D	7	2.1
[35]	Nir, 2014	Intensity- and landmark-based	AT	Yes	Manual landmarks	MRI/US	3D	10	3.8
[37]	Porter, 2001	Intensity-based: correlation	AT	Yes	Urethra	US	3D	3	2.4
[78]	Zhan, 2007	Intensity-based: mutual information	TPS	No	Manual landmarks	MRI	3D	5	1.5
[43]	Jo, 2008	Intensity-based: correlation	TPS	No	Root-mean-square manual landmarks	MRI	2D	4	1.5
[12]	Park, 2008	Intensity-based: mutual information	TPS [79]	Yes	Medial-axes tumour	MRI/PET	3D	2	3.0
[80]	Groenendaal, 2010	Intensity-based: correlation	BSp [81]	No	Manual (contour) landmarks	MRI	3D	5	2.2
[47]	Mazaheri, 2010	Intensity-based: binary similarity	FFD-BSp [82]	No	Surface overlap	MRI	2D	24	–
[83]	Chappelow, 2011	Intensity-based: mutual information	FFD-BSp [82]	No	Image similarity	MRI	2D	25	–
[84]	Patel, 2011	Intensity-based: spatially weighted mutual information	FFD-BSp [82]	No	Manual (contour) landmarks	MRI	2D	2	1.65
[38]	Orczyk, 2013	Intensity-based: mutual information	AT	No	3D volume overlap	MRI	3D	3	–
[44]	Kalavagunta, 2015	Intensity-based: ternary similarity	AT	No	Manual landmarks	MRI	2D	35	1.54
[18]	Reynolds, 2015	Intensity-based: normalized mutual information	FFD-BSp [82]	No	Manual landmarks	MRI	3D	6	3.1
[45]	Guzman, 2016	Intensity-based: mutual information	BSp [81]	No	Manual landmarks	MRI	2D	5	3.1
[50]	Shah, 2009	Mould-based	–	–	Visual inspection	MRI	–	–	–
[51]	Trivedi, 2012	Mould-based	–	–	visual inspection	MRI	–	1	–
[52]	Priester, 2014	Mould-based	–	–	Visual inspection	MRI	–	1	–
[55]	Starobinets, 2014	Mould-based	–	–	Manual landmarks	MRI	–	10	1.9
[53]	Elen, 2016	Mould-based	–	–	Manual ex vivo MRI landmarks	MRI	–	2^e^	0.92

^a^Most algorithms use a multi-step approach, usually starting with coarse rigid registration; only the last, most sophisticated registration step is mentioned

^b^*AT* affine transformation, *TPS* thin-plate spline, *(FFD)–BSp* (free form deformation)–basis-spline, *NN* natural neighbour

^c^# Number of prostates for the verification of the performance

^d^*TRE* target registration error

^e^Only two of the six prostates were used for verification

### Step 3: correlation

The final step is the transition from the registered three-dimensional models to the actual images. If the histopathology is directly registered to the ultrasonic modality under investigation, histopathologic voxels are easily mapped to imaging voxels or pixels (in which case, a model cross-section needs to be computed). For the evaluation of elastography or contrast-enhanced images and videos, the registration might have been performed to a three-dimensional or three-dimensionalized B-mode ultrasound first (as mentioned in Step 1B). Another registration step between B-mode and the final modality is then needed. Intra-modality registration could be performed along the same lines as in Step 2, or using fusion techniques as those mentioned in the Discussion [56].

## Discussion

Ultrasound imaging of the prostate is still rapidly advancing and especially promising modalities such as power Doppler [57], elastography [58], and contrast-enhanced ultrasound [59] have been extensively studied in recent years. Due to prostate deformation after excision, the effect of the transrectal probe, and the imaging planes not being parallel, histopathological validation using RP specimens is challenging.

**Fig. 3:**
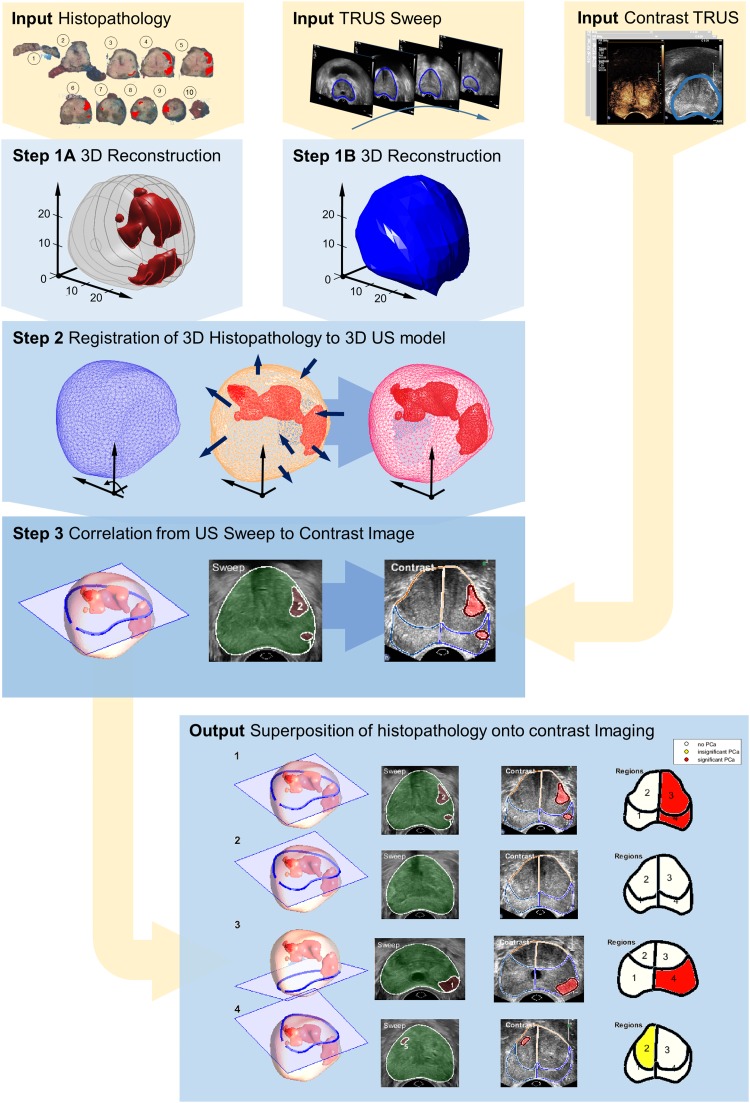
Schematic of an example registration framework for the correlation of the US image with histopathology; (1) 3D reconstruction of the ex vivo radical prostatectomy specimen and in vivo gland (2) registration between in vivo and ex vivo model; (3) correlation of the pathology data and the contrast-enhanced recording; (4) pixel-wise superposition of the histopathologic data

Based on our review of the literature, we have found that full histopathology-prostate registration is essentially a three-step process combining reconstruction, registration and correlation. For illustrative purposes, Fig. 3 depicts a possible procedure, matching contrast-enhanced ultrasound videos to histopathology. As no 3D imaging is available in this example, both a 3D histopathologic model [33] and a 3D in vivo model based on a B-mode US sweep are built. In this case, the models are subsequently registered using the method presented by Schalk et al. [13]. Now, by cognitively locating each imaging plane of the contrast recording in the B-mode sweep, histopathological cross-sections matching the contrast-ultrasound images can be computed. In a similar way, other reconstruction (Step 1) and registration (Step 2) techniques can be implemented and combined. We have found that most algorithms achieve an accuracy in the millimetre range. By employing an error margin between benign and malignant regions in the validation, one can take this into account.

Nonetheless, the question remains how small such a margin should be. Most papers stress that clinically significant tumours have radii exceeding 5 mm (having a cutoff volume of 0.5 cm^3^), deeming any registration error margin below that distance sufficient. However, it has been reported that around a quarter of PCa lesions are heterogeneous as to their Gleason Score [60], suggesting that sub-lesion accuracy is important to localize the high-grade PCa core. Fortunately, in 75% of the cases, the highest Gleason Score is found in the middle of the lesion [60]. In general, high-grade hotspots of heterogeneous significant tumours are considered to be 0.3 cm^3^ in median volume [61] and, therefore, we should actually aim for an error under 4.1 mm.

Prostate registration algorithms also play a vital role in fusion technology, in which the registration takes place between two imaging modalities [62]. Fusion generally finds its application in TRUS-guided biopsy procedures targeting suspicious areas found by another modality, in treatment planning for radiotherapy, or in the monitoring of a developing lesion over several sessions. For these purposes, respectively, recent literature features a wide range of variations of inter-modality fusion (e.g. PET–US [63], MRI–US [64], MRI–SPECT [65], and MRI–CT [66]) and intra-modality fusion techniques (e.g. US–US [67], CT–CT [68], and MRI–MRI [69]). Although fundamentally these algorithms can be extended for registration of imaging and histology, they are usually optimized for in vivo registration and do not have to cope with the large deformations typical for ex vivo specimens.

Clearly, registration is not the only source of inaccuracy in PCa validation. The quality of the imaging, segmentation, or (automatic) landmark detection affect the result as well. Prostate motion could also hinder the registration procedure, but, in contrast to MRI and PET, US acquisitions are generally sufficiently fast to avoid this. Displacements due to respiration, however, have been measured in the order of several millimetres and the use of transrectal equipment is known to stimulate muscular contractions [70]. This might severely affect ultrasound modalities with a longer acquisition time.

## Conclusion

It is important to be aware of the limitations and accuracy of registration techniques in PCa imaging. Unfortunately, implementation of full-registration procedures is still scarce in current PCa imaging studies. With the shift from PCa detection to PCa localization [71], however, such validation will be indispensable to study the imaging performance. In this review, the wide range of validation strategies has been discussed in the light of ultrasonic imaging. We also provided guidelines for registration and an example of a rigorous pixel-to-pixel matching procedure.

## List of abbreviations

2D: Two-dimensional
3D: Three-dimensional
AT: Affine transformation
BSp: Basis-spline
CT: Computed tomography
PCa: Prostate cancer
PET: Positron emission tomography
RBF: Radial-basis function
RP: Radical prostatectomy
TPS: Thin-plate spline
TRE: Target registration error
MRI: Magnetic resonance imaging
US: Ultrasound
